# Social isolation among indigenous college students in Peru: the role of language, culture, and acculturation

**DOI:** 10.3389/fsoc.2025.1597952

**Published:** 2025-06-23

**Authors:** Margit Julia Guerra Ayala, Carlos Antonio Valdivia Laura, Hania Nancy Bernedo Perez, Apolinar Florez Lucana, Rildo Raul Tapia Condori, Emma Lourdes Durand-Gómez

**Affiliations:** ^1^Universidad Tecnológica del Perú, Arequipa, Peru; ^2^Universidad Andina Néstor Cáceres Velásquez, Juliaca, Peru; ^3^Escuela de Educación Superior Pedagógica Arequipa, Arequipa, Peru

**Keywords:** adaptation, L2 competence, social isolation, cultures, Quechua, Aymara, psychological acculturation

## Abstract

Social isolation is a significant challenge faced by university students from indigenous Quechua and Aymara communities in Peru, often stemming from language barriers, cultural differences, and the pressure to assimilate into a predominantly Spanish-speaking academic environment. Isolation can negatively affect emotional wellbeing and academic success. This research aims to determine how psychological acculturation and Spanish L2 (second language) proficiency influence social isolation. The study included 202 university students from Quechua and Aymara communities in Peru (aged 18–30; 69.8% female, 30.2% male; 33.7% Quechua, 66.3% Aymara). A newly developed Psychological Acculturation Scale assessed cognitive and emotional adaptation processes, showing good reliability (ω = 0.774) and an adequate model fit in Confirmatory Factor Analysis, with CFI = 0.923, TLI = 0.899, SRMR = 0.0521, and RMSEA = 0.066. The Isolation and Loneliness Questionnaire (CAS), adapted for university students, was used to assess social isolation. Spanish L2 proficiency was evaluated using the CEFR (Common European Framework of Reference for Languages) descriptors. A non-experimental, quantitative design was employed, using simple linear regression to analyze the predictive power of psychological acculturation and Spanish L2 proficiency on social isolation. ANCOVA tested the moderating effects of gender, culture of origin, and academic performance. Results indicated that Spanish L2 proficiency strongly predicted social isolation, explaining 82.3% of the variance independently and 85.6% when combined with psychological acculturation. Interaction effects between gender, cultural background, and academic performance were not significant. This study provides evidence for the crucial role of language proficiency in the social integration and academic success of indigenous students, calling for targeted interventions that address linguistic barriers and promote cultural understanding.

## 1 Introduction

In today's global landscape, cultural interconnectedness shapes the experiences of individuals worldwide (Walkowitz et al., 2022). Accelerated globalization has given rise to the complex and inevitable phenomenon of acculturation, a process that involves the integration of individuals into new cultural contexts and triggers fundamental psychological transformations (Peng et al., 2023). Language, in this context, is not only a communication tool but also a vehicle for cultural negotiation and integration (Berry, 2006, as cited in Ogungbe et al., 2022). However, integration often faces obstacles such as language barriers, unfamiliar academic systems, and sociocultural mismatches that hinder adaptation (Kayama and Yamakawa, 2020; Dornyei and Ryan, 2015).

In the Peruvian context, systemic inequalities within the education system reflect deep-rooted historical and structural barriers (Huaman and Valdiviezo, 2014). These include the longstanding marginalization of Indigenous languages and cultures, the limited availability of intercultural bilingual education in higher education institutions, and the centralized design of academic curricula that often overlooks the sociocultural realities of Indigenous populations (Hidalgo Collazos, 2025). Such conditions perpetuate educational disadvantage and hinder equitable access to resources and opportunities for Quechua and Aymara students (Ortiz-Melgar, 2022; García-Segura, 2017). Spanish, as the dominant academic language in Peru, becomes a key gateway—or barrier—for social inclusion (Mackie and Monroy, 2022). Students with limited L2 proficiency often face increased acculturative stress and social isolation (Martirosyan et al., 2015).

Social isolation in this context refers to the subjective experience of disconnection, loneliness, and exclusion, often linked to low perceived social support and a lack of belonging (Caldas and Silva, 2024; Durat et al., 2023). These psychosocial challenges are particularly evident among Indigenous students from rural backgrounds who migrate to urban academic settings, where the dominant language and culture differ significantly from their own.

Previous studies have shown that factors such as gender, cultural background, and academic performance may influence students' adaptation experiences and levels of social isolation. Gender differences have been documented in relation to perceived social disconnection, with women often reporting greater isolation in academic environments (Rodrigues et al., 2021). Similarly, cultural background can shape identity salience and acculturative stress, particularly among students from Indigenous populations such as the Aymara and Quechua (García-Segura, 2017). Additionally, academic performance may affect students' sense of belonging and self-efficacy, where academic difficulties can increase the risk of marginalization (Selkova, 2020).

Therefore, the general objective of this study is to determine the extent to which psychological acculturation and Spanish language proficiency explain the variability of social isolation among Peruvian university students from Quechua and Aymara Indigenous cultures in a more urban academic environment. The specific objectives aim to determine whether there is a significant association between the acculturation process, the dominant language, and social isolation, also considering the possible moderating effects of factors such as gender, cultural background, and academic performance.

### 1.1 Literature review

In Peru, the Quechua and Aymara peoples constitute the two largest Indigenous ethnolinguistic groups. According to national census data, ~22.3% of the Peruvian population identifies as Quechua and around 2.4% as Aymara [Instituto Nacional de Estadística e Informática (INEI), 2018]. These communities are predominantly located in the Andean highlands of southern Peru, in regions such as Puno, Cusco, Apurímac, and Ayacucho.

Historically, both groups have endured prolonged cultural and linguistic marginalization since the Spanish colonial period, which imposed Castilian as the dominant language and actively suppressed Indigenous languages, identities, and worldviews. This process continued through successive republican governments, where Spanish-centric educational policies systematically excluded Indigenous knowledge and linguistic rights from the national curriculum (Hornberger, 2011). Although Peru is now officially recognized as a multicultural and multilingual country, and intercultural bilingual education has been promoted in basic education, higher education remains predominantly monolingual and monocultural (García-Segura, 2017). Indigenous students, especially those from rural Andean backgrounds, often encounter an academic system that does not reflect or validate their cultural identities, generating significant challenges of adaptation and belonging (Ortiz-Melgar, 2022). This mismatch is further exacerbated in urban academic environments, where institutional support for linguistic or cultural integration is minimal.

These structural and symbolic barriers contribute not only to educational inequality but also to the persistence of epistemic injustice, whereby Indigenous students must assimilate into dominant discourses and suppress their linguistic-cultural heritage in order to succeed academically (de Sousa Santos, 2017). Understanding this historical and sociopolitical context is essential to interpreting the psychological and social dynamics that Indigenous students experience in higher education.

### 1.2 Social isolation

Social isolation is a multifaceted psychological and sociological construct, defined by a subjective sense of disconnection from others, low perceived social support, and the absence of meaningful interpersonal relationships (Cornwell and Waite, 2009, as cited in Suwinyattichaiporn and Johnson, 2022; Caldas and Silva, 2024). It differs from objective measures of social contact by emphasizing the perception of isolation, which can occur even in the presence of others.

In university settings—particularly those involving intercultural transitions—social isolation can be exacerbated by language barriers, cultural unfamiliarity, and systemic exclusion (Myles and Cheng, 2003; Schreuders et al., 2023). Its impact is not only emotional—manifesting as loneliness, depression, and anxiety (Decety and Cacioppo, 2011)—but also cognitive and behavioral, reducing academic engagement, motivation, and performance (Kemelgor and Etzkowitz, 2001).

Gender and ethnic background have also been identified as risk factors for isolation in academic and professional environments (Martin-Storey et al., 2021). Women, especially those from historically marginalized communities, are more likely to report higher levels of isolation due to discrimination, lack of representation, and difficulties accessing support networks (Rodrigues et al., 2021; Vézina et al., 2004). Among Indigenous university students, social isolation is intensified by linguistic and cultural dissonance with the institutional environment, leading to emotional distress and reduced persistence in higher education, as demonstrated in other contexts (Walton et al., 2020).

This phenomenon of social isolation is closely intertwined with acculturation processes, particularly in internal migration contexts, where cultural adaptation directly influences students' perceived social integration.

### 1.3 Acculturation

Acculturation, language, and social isolation have been extensively studied in international contexts, where students face significant pressures to adapt culturally, linguistically, and academically after migrating to foreign countries (Benson, 2016). However, similar phenomena also arise in multilingual and multicultural societies with high internal mobility, such as Peru, where Indigenous students migrate from rural areas to urban centers (García-Segura, 2017; Mamani-Vilchez et al., 2021). In such settings, adaptation transcends the linguistic and academic dimensions and involves deep psychological transformations at the individual level. These transformations lead to a type of acculturation that goes beyond functional integration—such as adherence to basic social norms or participation in academic or work-related activities (Kamalova et al., 2020; Wilczewski and Alon, 2023)—and instead involves internal processes linked to thought, identity, and emotional experience.

This form of acculturation includes both cognitive and emotional processes that take place at the individual level during cultural adaptation (Deng et al., 2025). The cognitive dimension refers to changes in the way people think about their culture of origin and the host culture, while the emotional dimension encompasses factors such as belonging, cultural identity, and the emotional stress associated with adaptation (Yoon et al., 2012).

The cognitive dimension of acculturation involves an internal process of mental adaptation that occurs in immigrant individuals or in contexts of intercultural contact. This process includes various subprocesses that transform how individuals understand and relate to their environment (Sam and Berry, 2016). Each person develops a worldview shaped by their upbringing and sociocultural context, which guides how they interpret the world. When faced with an acculturation process, this worldview is challenged by exposure to new values, norms, ideas, and ways of thinking from the host culture. This encounter may prompt a deep reevaluation of one's beliefs, attitudes, and prior perspectives about the self and the surrounding world (Berry et al., 2002), leading to transformations such as the adoption of new reasoning frameworks or the integration of multiple cultural perspectives. These shifts can cause internal conflict and stress when individuals confront contradictory norms, potentially affecting academic participation, socialization (Morita, 2009), and subjective wellbeing (Yoon et al., 2012). Nevertheless, as the acculturation process advances, individuals often develop greater cognitive flexibility and adaptive capacity (Guzel, 2016) including the adoption of new ways of thinking and potential adjustments to personal identity (Sam and Berry, 2016). These changes do not necessarily imply a loss of the original identity, but rather an evolution or expansion in response to new influences, often preserving a connection to one's cultural heritage (Benson, 2016).

The cognitive component also involves understanding and adopting cultural customs and beliefs—actively acquiring knowledge and accepting the practices and values of the host culture (Lee, 2007). In university contexts, student engagement and the cultural openness of faculty are essential for building inclusive educational spaces (Devereaux et al., 2010). In addition, social support plays a key role in reducing acculturative stress (Kristiana et al., 2022; Martirosyan et al., 2015). Intellectual understanding thus requires ongoing learning and an active pursuit of knowledge about the host culture's history, values, and traditions (Caldas and Silva, 2024).

Beyond cognitive processes, psychological acculturation also involves an emotional dimension (De Leersnyder, 2017). People often maintain a deep emotional attachment to their culture of origin, which constitutes a core element of their identity and significantly influences their adaptation to the new environment (Vishnevskaya, 2020). During cultural adjustment, it is common to experience longing for familiar elements—such as traditions, customs, foods, and social relationships—which can generate sadness and nostalgia for what has been left behind (Sokolova et al., 2022). In this sense, nostalgia becomes an emotional response that may emerge when a person is immersed in a new culture (Zou and Petkanopoulou, 2022). However, persistent nostalgia for the culture of origin may indicate that the individual is facing substantial difficulties adapting to the host culture (Ali et al., 2021). This may stem from a lack of connection with the new cultural environment, challenges in social integration, or a sense of alienation in the new context (Nguyen et al., 2019).

These findings reinforce the need to approach social isolation from an intersectional perspective that recognizes the role of language and acculturation as central psychological mechanisms in the university experience of Indigenous students.

## 2 Materials and methods

This study employed a non-experimental quantitative design with a correlational, comparative, and explanatory approach.

### 2.1 Participants

The participants in this study were college students who had migrated to the city of Arequipa, Peru. A total of 202 students were selected through snowball sampling and identified based on their cultural origin (Aymara or Quechua) through recommendations from other students. The sample comprised 61 male students (30.2%) and 141 female students (69.8%) aged between 18 and 30. Regarding cultural background, 68 students identified as Quechua, while 134 identified as Aymara.

Inclusion criteria required that participants (a) be enrolled in an undergraduate program in Arequipa, (b) self-identify as belonging to the Quechua or Aymara Indigenous groups, (c) be between 18 and 30 years old, and (d) possess at least basic functional proficiency in Spanish, as determined by their ability to complete self-report questionnaires and engage in academic activities in Spanish. Exclusion criteria included: (a) being under 18 years of age, (b) lack of sufficient Spanish language skills to comprehend and respond to the instruments, or (c) non-enrollment in university-level studies at the time of the research. All participants were actively enrolled in undergraduate programs, which was an inclusion criterion. Participants had migrated from rural areas to the city of Arequipa between 2020 and 2023, with most of them having resided in the city for at least one academic year at the time of participation. Informed consent was obtained from all participants before completing the instruments, ensuring their voluntary participation and confidentiality.

### 2.2 Instruments

For the purposes of this study, three instruments were used, the first of which was constructed specifically for this research: the *Psychological Acculturation Scale* (PAS-10). This scale assesses the cognitive and emotional aspects of psychological acculturation and consists of 10 items−5 corresponding to the cognitive dimension and 5 to the emotional dimension. Each item is responded to using a three-point Likert scale, allowing participants to indicate the degree to which they agree with each statement: “agree,” “neither agree nor disagree,” and “disagree.” In the cognitive domain, the items explored changes in thinking patterns when adapting to a new culture, the ability to understand and assimilate the cultural norms of the current environment, and the evolution of cultural identity since the move. Items were also considered with content about the assessment of the importance of understanding and adopting the customs and beliefs of the host culture, as well as maintaining constant learning about that culture's history, values, and traditions. Regarding emotional aspects, the instrument measures the persistence of nostalgia for the culture of origin, emotional connection with people in the new environment, emotional satisfaction with cultural adaptation, and enthusiasm for cultural differences. Item 6, which belongs to the emotional dimension, was reverse-coded due to its negative formulation. The complete *Psychological Acculturation Scale* (PAS-10), including all items, is available in the Supplementary material.

The second instrument used in this study was the Cuestionario de Aislamiento y Soledad (CAS), a self-report instrument originally developed to assess feelings of loneliness and social isolation. The scale consists of 25 items−12 measuring loneliness and 13 measuring social isolation—rated on a four-point Likert scale (always, often, sometimes, never). Although the original version was designed by Casullo in 1996, its structure and use are described by Tapia et al. (2003), who applied the instrument in a sample of Argentine adolescents. In that study, the CAS showed sensitivity to differences in perceived isolation levels based on percentile rankings and revealed a strong negative association between loneliness-isolation scores and self-concept indicators. Although originally used with adolescents, the CAS was considered appropriate for this study given the relevance of its underlying constructs—loneliness and perceived social disconnection—for Indigenous university students navigating cultural and academic transitions. For the present study, the CAS was linguistically reviewed and contextually adapted for use with this population. Given the differences in population and cultural context, we conducted a psychometric reanalysis to evaluate internal consistency and model fit in this new sample. In this sample, the adapted CAS demonstrated acceptable internal consistency, with a Cronbach's alpha of 0.83.

The third instrument employed in this study was a rubric-based assessment of Spanish language proficiency, aligned with the Common European Framework of Reference for Languages (CEFR). A linguistics specialist from the research team conducted the evaluation, assessing the four key communicative skills: listening comprehension, reading comprehension, speaking, and writing. The assessment was carried out through a combination of standardized tasks and spontaneous language production samples. For example, participants listened to short dialogues between students discussing academic challenges (e.g., “What is the main issue they are trying to solve?”) or university announcements (e.g., “What event is being announced, and who is it for?”). Reading tasks included comprehension of personal letters and informational texts related to student life (e.g., “What is the student's main feeling?” or “How can the support service be accessed?”). Speaking activities included describing a personal tradition (“Describe a tradition from your home region and explain its meaning”) or comparing their previous school with their current university experience. Writing tasks asked participants to produce short opinion or descriptive texts, such as “Do you think speaking Spanish helps you feel integrated in class? Explain why” or “Describe your first week at university and how you felt about it.” The rubric followed CEFR performance descriptors, focusing primarily on A2 to B2 levels. This approach ensured a valid and integrative assessment of communicative competence, in line with recommendations by Carbó Marro (2011), who emphasized the importance of evaluating academic language performance in multilingual learners. The evaluation was objective and carried out individually by the linguist, not based on self-assessment. The CEFR descriptors used to evaluate Spanish language proficiency are available in the Supplementary material.

### 2.3 Procedures

Following the inclusion and exclusion criteria, the research team identified an initial group of participants through targeted outreach directed at Quechua- and Aymara-speaking university students enrolled in higher education institutions in Arequipa. This outreach was facilitated through peer networks, student groups, and academic staff who had regular contact with Indigenous students. Given that two members of the team have family ties to the Aymara and Quechua cultures, a culturally appropriate recruitment process was ensured. These researchers were personally familiar with the initial participants, which helped establish trust and facilitated the first phase of recruitment. Furthermore, initial participants were encouraged to share the study details with their peers, thereby expanding recruitment through culturally resonant and trusted social circles.

The first two instruments, self-administered in person, were completed in an average of 50 min. Both included informed consent, clearly stating that participation was entirely voluntary. The researchers emphasized that participants could choose not to participate or not to complete the instruments without facing any consequences. This respect for their autonomy and the assurance that their responses would remain confidential and be used exclusively for research purposes created a safe environment, fostering honest and open participation. To ensure participant confidentiality, a unique code was assigned to each participant, which was used to link their responses across the different instruments. No information was collected that would allow direct identification of the participants. The third instrument, a rubric, was administered with an approximately average duration of 20 min per participant. The researchers collected data from March to December 2024.

### 2.4 Analysis of the data

This study employed several statistical models to analyze the relationships among psychological acculturation, Spanish L2 proficiency, and social isolation, considering also other intervening factors. The analysis applied three main models: multiple linear regression, correlation, and analysis of covariance (ANCOVA). Correlation analyses were conducted to explore the associations between psychological acculturation and social isolation, as well as between Spanish L2 proficiency and social isolation, quantifying the magnitude and direction of the relationships without establishing causality. Multiple linear regression was performed in two stages: first, the impact of Spanish L2 proficiency on social isolation was evaluated, and then this model was compared with a second model that included both Spanish L2 proficiency and psychological acculturation to assess the combined impact of both independent variables. The ANCOVA model examined how psychological acculturation and Spanish L2 proficiency influence social isolation, considering variables such as gender, culture (Aymara and Quechua), and academic performance, allowing to evaluate if the effect varies according to these factors. Prior to conducting the analyses, key assumptions for parametric testing were verified. Normality of residuals was supported by the Shapiro-Wilk test (*W* = 0.993, *p* = 0.440), Kolmogorov-Smirnov test (*D* = 0.059, *p* = 0.482), and Anderson-Darling test (*A*^2^ = 0.362, *p* = 0.440). Linearity and homoscedasticity were assessed through residual plots and confirmed by the Goldfeld-Quandt (*F* = 1.02, *p* = 0.466) and Harrison-McCabe tests (*H* = 0.494, *p* = 0.428), although the Breusch-Pagan test indicated heteroskedasticity (χ^2^ = 34.9, *p* < 0.001). Autocorrelation was ruled out (Durbin-Watson = 1.89, *p* = 0.436). Additionally, no missing data were identified, and multicollinearity was within acceptable limits (VIFs < 1.30). The statistical package used was Jamovi 2.3.21.

## 3 Results

Before examining the predictive models, the psychometric properties of the newly developed PAS-10 were assessed to ensure the reliability and validity of the instrument used in this study. The instrument demonstrated acceptable internal consistency, with a McDonald's omega coefficient of 0.774 (Table 1).

**Table 1:** Reliability scale statistics.

**Scale**	**McDonald's omega (ω)**
PAS-10 scale	0.774

McDonald's omega (ω) is a reliability coefficient that measures a scale's internal consistency, providing an alternative to Cronbach's alpha.

Table 2 presents additional measures from the instrument validation process. The CFI and TLI compare the fit of the proposed model with a null model, with values ranging from 0 to 1. Closer to 1 indicates a better fit, with values >0.90 considered adequate and more significant than 0.95 excellent. In this case, the CFI is 0.923, and the TLI is 0.899, suggesting a good and acceptable fit. The SRMR measures the average discrepancy between observed and estimated correlations in the model, where values below 0.08 are considered good. Here, the SRMR is 0.0521, indicating a perfect fit. The RMSEA measures the model fit of the approximation error in the population. Values below 0.05 are good, between 0.05 and 0.08 acceptable, and above 0.10 deficient. Here, the RMSEA is 0.0667, within the acceptable range. The confidence intervals of the RMSEA provide an estimate of its accuracy, with a narrower interval indicating better precision.

**Table 2:** Fit measures.

**CFI**	**TLI**	**SRMR**	**RMSEA**	**90% CI of RMSEA**	
				**Inferior**	**Superior**
0.923	0.899	0.0521	0.0667	0.0413	0.0913

Fit indices indicate the goodness-of-fit for the model. CFI, Comparative Fit Index; TLI, Tucker-Lewis Index; SRMR, Standardized Root Mean Square Residual; and RMSEA, Root Mean Square Error of Approximation, with 90% confidence intervals are reported.

**Figure 1:**
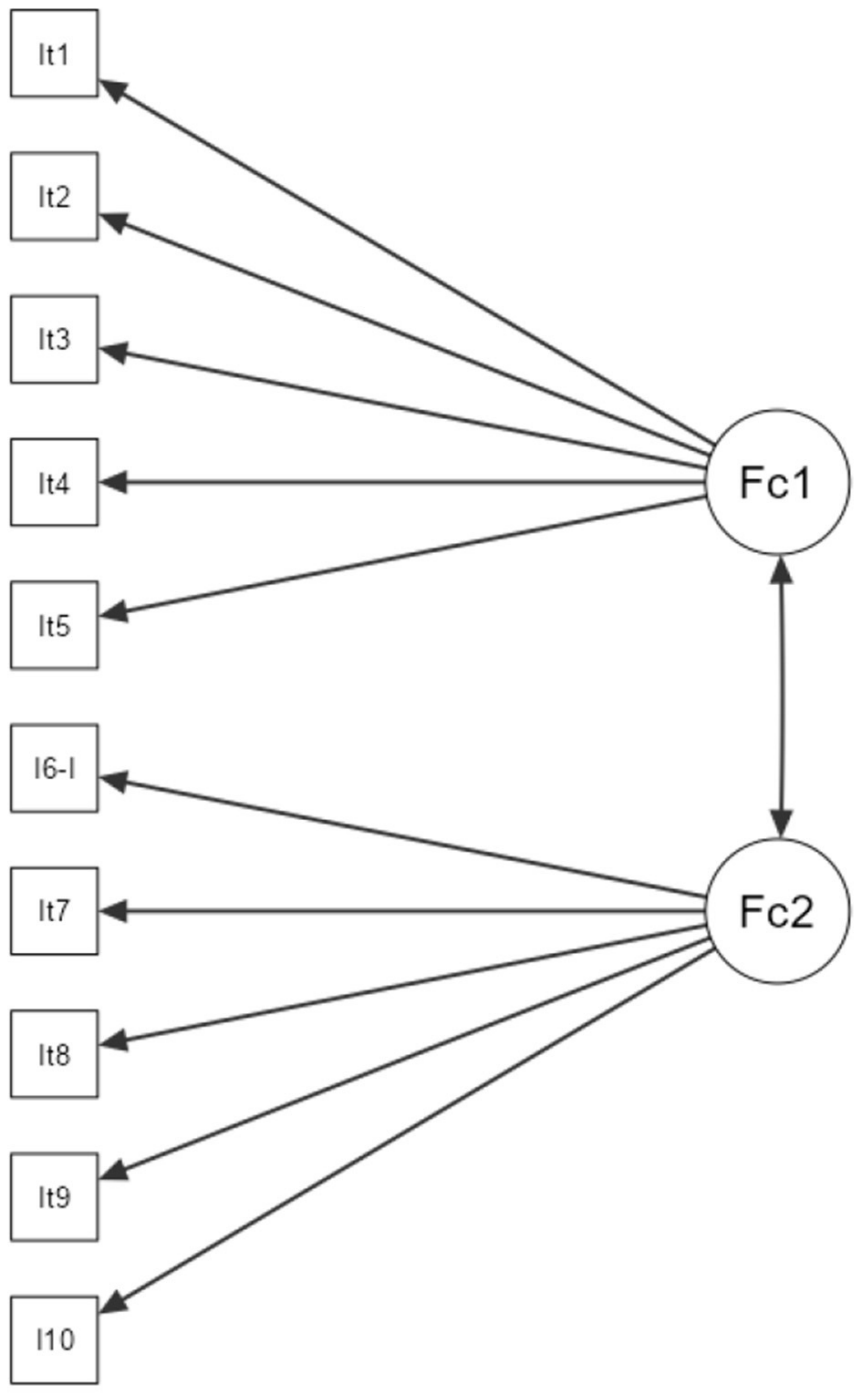
Psychological acculturation scale (PAS-10). The figure displays the analysis flow of the PAS-10 instrument, revealing two factors and their interactions.

Figure 1 illustrates the statistical analysis's flow of instrument items (PAS-10). The “Minimum Residual” extraction method was combined with an Oblimin rotation to identify the instrument's underlying dimensions. The results indicated a two-factor structure that aligned with the theoretical hypotheses. These factors are cognitive psychological acculturation and emotional psychological acculturation.

The adapted version of the CAS used in this study yielded a Cronbach's alpha of 0.71, a CFI of 0.91, and an RMSEA of 0.07, indicating acceptable reliability and model fit (see Tables 3, 4).

**Table 3:** Model fit measures.

**Model**	***R***	***R* ^2^**	**Adjusted *R*^2^**	**Overall model test**			
				***F***	**df1**	**df2**	***p*** **-value**
1	0.907	0.823	0.821	463	2	199	<0.001
2	0.925	0.856	0.854	392	3	198	<0.001

Models estimated using sample size of N = 202. High and significant F-values (*p* < 0.001) suggest that the models are statistically significant.

**Table 4:** Omnibus ANOVA test.

**Source**	**Sum of squares**	**df**	**Mean square**	***F***	***p*-value**
Spanish L2 proficiency	223.4	2	111.708	254.5	<0.001
Psychological acculturation	19.8	1	19.770	45.0	<0.001
Residuals	86.9	198	0.439		

Type 3 sum of squares is appropriate for models with interactions or when it is desired to evaluate the main effect of a factor in the presence of other factors.

Two linear regression models were conducted to analyze the impact of Spanish L2 proficiency and psychological acculturation on social isolation within Quechua and Aymara communities and compare the explanatory power of each variable.

Table 3 presents both models, which are statistically robust and have considerable explanatory power. Model 1 considers Spanish L2 proficiency as a predictor variable and social isolation as a dependent variable, with a coefficient of determination (*R*^2^) of 0.823. That indicates that ~82.3% of the variance in social isolation can be explained by Spanish L2 proficiency. On the other hand, Model 2 incorporates both linguistic competence as a factor and psychological acculturation as a covariate. This model explains an even more significant percentage of variance (*R*^2^ = 0.856), indicating that 85.6% of the variability in social isolation can be attributed to the interaction of both predictor variables.

Both models present significant *F*-values [*F*_(463, 392)_], which support the validity of the proposed models. The minimal difference between *R*^2^ and adjusted *R*^2^ in both models suggests that the fit is not overfitted, reinforcing the results' robustness and minimizing the risk of error due to unnecessary complexity in the model.

The Omnibus ANOVA test applied in the linear regression (Table 4) determined that the model, which includes Spanish L2 proficiency as a factor and psychological acculturation as a covariate, explains a significant proportion of the variance in the dependent variable, social isolation. The statistical significance of the model (*p* < 0.05) validates that both Spanish L2 proficiency and the processes of psychological acculturation are key elements in predicting social isolation. These results elucidate that the model is statistically robust and an effective predictive tool.

Table 5 shows that the inclusion of psychological acculturation as a predictor variable significantly improves the explanation of social isolation. Incorporating this covariate in Model 2 increases the explanatory power by 3.31% compared to Model 1 (Δ*R*^2^ = 0.0328), which represents a relevant advancement in understanding the factors that contribute to isolation. Furthermore, including psychological acculturation significantly improves the model's fit, as indicated by the *F* test (*F* = 45.0, *p* < 0.001). That suggests that psychological integration into the dominant culture has a statistically significant impact on levels of social isolation. These results highlight that linguistic proficiency in L2 influences social isolation, and levels of psychological acculturation play a determining role.

**Table 5:** Model comparison.

**Comparison**		**Δ*R*^2^**	***F***	**df1**	**df2**	***p***
**Model**	**Model**					
1	2	0.0328	45.0	1	198	<0.001

Table 6 presents the coefficient values of the model, which reflect the differences in the dependent variable, social isolation, between the different levels of the categorical variables, taking the highest level of each category as a reference. The coefficient corresponding to the “Low–High” comparison (3.0979) for Spanish L2 proficiency reveals that individual with low linguistic proficiency experience significantly higher levels of social isolation than those with high competence. Similarly, the coefficient for “Moderate–High” (1.5526) indicates that, although to a lesser extent, those with moderate linguistic competence also exhibit greater social isolation than those with high proficiency. As for psychological acculturation, the coefficient of −0.0878 suggests that for each unit increase in this process, the level of social isolation significantly decreases (*p* < 0.001), even when keeping linguistic competence constant. Its statistical relevance confirms that psychological acculturation acts as a protective factor against isolation.

**Table 6:** Model coefficients—social isolation.

**Predictor**	**Estimator**	**SE**	***t***	***p*-value**
Constant^a^	6.6502	0.3815	17.43	<0.001
**Spanish L2 proficiency**				
Low–high	3.0979	0.1375	22.54	<0.001
Moderate–high	1.5526	0.1259	12.33	<0.001
Psychological acculturation	−0.0878	0.0131	−6.71	<0.001

a Represents the reference level.

A correlation analysis was conducted because the model coefficients do not always reflect direct correlations or adequately capture the linear relationship between a categorical variable (such as Spanish L2 proficiency) and the dependent variable (social isolation). In this case, Kendall's Tau-b coefficient, suitable for evaluating associations between ordinal variables, was used. The results, presented in Table 7, show a very strong inverse correlation between linguistic competence and social isolation (Tau-b = −0.880, *p* < 0.001). This negative association indicates that as the level of linguistic competence in L2 increases, the levels of social isolation tend to decrease significantly.

**Table 7:** Correlation matrix.

**Variables**	**Coefficients**	**Spanish L2 proficiency**	**Social isolation**
Spanish L2 proficiency	Kendall's Tau B	–	
	*p*-value	–	
Social isolation	Kendall's Tau B	−0.880^***^	–
	*p*-value	<0.001	–

* *p* < 0.05,

** *p* < 0.01,

*** *p* < 0.001.

Table 8 presents the results of the correlation between psychological acculturation and social isolation, revealing a significant inverse relationship between both variables (*r* = −0.678, *p* < 0.001). This negative association suggests that as individuals achieve greater psychological integration with the dominant culture, levels of social isolation decrease considerably. The strength of this relationship indicates that psychological acculturation is not only a relevant factor but could also play a protective role in preventing social isolation. Individuals who internalize cultural elements of their environment tend to establish stronger social connections, facilitating their adaptation and reducing feelings of exclusion. These results underscore the importance of promoting cultural integration strategies that go beyond linguistic learning and addressing psychological adaptation as an essential component to reducing social isolation in multicultural contexts.

**Table 8:** Correlation matrix.

**Variables**	**Coefficients**	**Psychological acculturation**	**Social isolation**
Psychological acculturation	Rho de Spearman	–	
	df	–	
	*p*-value	–	
	*N*	–	
Social isolation	Rho de Spearman	−0.678^***^	–
	df	200	–
	*p*-value	<0.001	–
	*N*	202	–

^*^p < 0.05, ^**^p < 0.01, ^***^p < 0.001.

The results presented in Table 9 indicate that the most determining factors in the level of social isolation are psychological acculturation (*F* = 37.64, *p* < 0.001) and Spanish L2 proficiency (F = 146.20, *p* < 0.001). These results highlight the importance of cultural integration processes and language proficiency as key variables that influence the reduction of isolation. In contrast, variables such as sex, culture of origin, academic performance, and their possible interactions do not show statistically significant effects on social isolation. That suggests that, although these factors could influence other aspects of social adaptation, their direct impact on isolation is minimal in this sample. The data support the idea that adaptation to a new cultural environment and language proficiency are the central elements that facilitate social inclusion. Cultural integration processes and linguistic competence are thus configured as the central modulators of social isolation, above other sociodemographic or academic variables.

**Table 9:** ANCOVA—social isolation.

**Model terms**	**Sum of squares**	**df**	**Mean square**	***F***	***p*-value**
Global model	159.26571	36	4.42405	33.5121	<0.001
Sex	0.07106	1	0.07106	0.1616	0.688
Culture	0.00510	1	0.00510	0.0116	0.914
Academic performance	2.66959	2	1.33479	3.0362	0.051
Psychological acculturation	16.54879	1	16.54879	37.6430	<0.001
Spanish L2 proficiency	128.54650	2	64.27325	146.2004	<0.001
Sex ✻ Culture	0.03653	1	0.03653	0.0831	0.774
Sex ✻ Academic performance	0.82111	2	0.41056	0.9339	0.395
Culture ✻ Academic performance	0.43076	2	0.21538	0.4899	0.614
Sex ✻ Spanish L2 proficiency	0.34042	2	0.17021	0.3872	0.680
Culture ✻ Spanish L2 proficiency	2.09666	2	1.04833	2.3846	0.095
Academic performance ✻ Spanish L2 proficiency	2.28709	4	0.57177	1.3006	0.272
Sex ✻ Culture ✻ Academic performance	0.08622	2	0.04311	0.0981	0.907
Sex ✻ Culture ✻ Spanish L2 proficiency	0.32442	2	0.16221	0.3690	0.692
Sex ✻ Academic performance ✻ Spanish L2 proficiency	1.74365	4	0.43591	0.9916	0.414
Culture ✻ Academic performance ✻ Spanish L2 proficiency	0.93832	4	0.23458	0.5336	0.711
Sex ✻ Culture ✻ Academic performance ✻ Spanish L2 proficiency	2.31949	4	0.57987	1.3190	0.265
Residuals	72.53800	165	0.43962		

## 4 Discussion

This study examined the influence of psychological acculturation and linguistic proficiency in Spanish, the dominant language in the university context, on the levels of social isolation among students. The findings offer a detailed perspective on the influence of psychological acculturation and linguistic proficiency on social isolation within the studied university context. Subsequently, the results obtained will be explored in greater depth, analyzing how psychological acculturation and linguistic proficiency relate to social isolation, and the implications of these findings within the university context will be discussed. Additionally, potential explanations for the lack of significance of other factors analyzed, such as sex, culture, and academic performance, will be explored, and the results will be compared with previous research in the field.

Regarding the first finding, linguistic proficiency in Spanish proved to be a significant predictor of social isolation, explaining 82.3% of the variance in this variable. This result underscores the importance of considering L2 competence as a crucial factor in understanding social isolation in individuals adapting to a new environment, especially in bilingual communities such as Quechua and Aymara. Psychological acculturation, although also significant, explained a smaller percentage of the variance (85.6% in conjunction with linguistic competence), suggesting that the ability to communicate effectively in the dominant language plays a primary role in social integration in these contexts. Competence in L2 plays a fundamental role in mitigating social isolation, particularly in multicultural academic settings where language proficiency directly influences communication and social integration (Saito et al., 2018). Limited proficiency in L2 often exacerbates feelings of exclusion and hinders meaningful participation in academic and social interactions (Yu et al., 2025). Studies highlight that linguistic barrier not only contribute to academic challenges but also reduce opportunities to build social networks (Yeh and Inose, 2003), the risk of isolation (Yeh and Inose, 2003). For indigenous students from linguistically diverse backgrounds, the development of strong L2 skills can foster greater inclusion and help bridge the cultural gap in higher education settings (Durán and Palmer, 2014).

Based on the model's coefficients, it was shown that psychological acculturation has a protective effect against social isolation. Specifically, it was observed that for each unit increase in psychological acculturation, the level of social isolation significantly decreases (*p* < 0.001), even while keeping L2 competence constant. This result is consistent with the theory that psychological acculturation facilitates social integration and establishes connections within the new community (Arenas and Urzúa, 2015, cited in Mamani-Vilchez et al., 2021). However, it is important to highlight that this acculturation process does not necessarily imply a loss of the original cultural identity. Instead, it can lead to an evolution or expansion of the sense of identity in response to new influences and experiences (Wang et al., 2016). Understanding and adopting the customs and beliefs of the cultural environment is a crucial component of this process, as it involves not only knowledge but also the active integration of these practices into daily life (Kristiana et al., 2022).

The model coefficients presented significant differences in social isolation among the various levels of linguistic competence in Spanish, the dominant language, and psychological acculturation. Consistent with previous research, low competence in the dominant language or L2, like in our case Spanish, is observed to be associated with significantly higher levels of social isolation compared to high competence (Martirosyan et al., 2015; Ogungbe et al., 2022). This underscores the importance of L2 proficiency as a key protective factor against isolation, especially in multicultural settings where mastery of the dominant language facilitates social integration and participation in academic and social interactions (Zorlu and Hartog, 2018). Beyond its communicative utility, language operates as a cultural vehicle that enables individuals to access shared meanings, practices, and values, thereby fostering a sense of belonging. As such, linguistic proficiency not only supports academic success but also serves as an integrative force that mitigates anxiety and social isolation (Xethakis et al., 2024). Similarly, although to a lesser extent, moderate competence in Spanish is also associated with greater isolation compared to high competence. Regarding psychological acculturation, the results indicate that for each unit increase in this process, the level of social isolation decreases significantly (*p* < 0.001), even when controlling for competence in Spanish. This finding supports the notion that psychological acculturation acts as a protective factor against isolation, facilitating social integration and the establishment of connections within the new community (Kristiana et al., 2022). Taken together, these results highlight the importance of addressing both linguistic barriers in L2 and cultural adaptation processes in interventions aimed at reducing social isolation in diverse contexts.

The results also confirmed the existence of a significant inverse relationship between L2 competence and social isolation. This finding aligns with recent research highlighting the importance of linguistic competence in social integration. For example, a study on reading competence in bilingual communities suggests that proficiency in multiple languages facilitates better adaptation and social cohesion (Mantilla Falcón et al., 2022). Additionally, research in multicultural educational settings indicates that acquiring the dominant language of the school environment among immigrant students not only improves their academic performance but also fosters greater social interaction and reduces feelings of isolation (Álvarez-Sotomayor and Martínez-Cousinou, 2020). These results emphasize the need to implement educational programs that strengthen L2 competence, not only as an academic tool but also as a means to promote inclusion and social wellbeing in multicultural contexts.

The findings furthermore reinforce the role of psychological acculturation in reducing social isolation, in line with contemporary research that emphasizes the importance of cultural integration beyond mere linguistic competence (Girmay and Singh, 2019). The significant inverse correlation found suggests that individuals who achieve higher levels of psychological acculturation experience lower levels of social isolation. This relationship has been consistently reported in studies on immigrant adaptation, where the internalization of cultural norms and values fosters a sense of belonging and strengthens social ties (Hawkins et al., 2021). Moreover, recent research indicates that psychological acculturation enhances emotional wellbeing and reduces the stress associated with cultural displacement, thereby preventing social withdrawal (Benet-Martínez and Repke, 2020). It is important to highlight those interventions promoting cultural integration, such as mentoring programs and community engagement initiatives, have been shown to facilitate social connections and improve psychological adaptation (Nguyen et al., 2019).

The analysis obtained through the ANCOVA test revealed that gender, cultural background (Aymara or Quechua), and academic performance do not significantly affect social isolation in this model, and the interactions between these variables are also not significant. In contrast, various studies have indicated that sociodemographic factors, such as access to social and economic resources, significantly influence individuals' adaptation and, consequently, their level of social isolation (Benet-Martínez and Repke, 2020; Ward et al., 2021). According to Selkova (2020), students in multicultural environments face adaptation challenges resulting from psychological acculturation, a process that can generate internal conflicts and stress due to the need to reconcile cultural differences between their place of origin and their host environment (Girmay and Singh, 2019).

Unlike the findings of the aforementioned studies, our results suggest that variables such as gender and cultural background do not play a determining role in social isolation within this model. While previous research has indicated that women tend to experience greater social isolation in academic and professional settings (Rodrigues et al., 2021), the data obtained in this study do not show significant gender-based differences. Similarly, studies on Indigenous communities have suggested that cultural background may represent an additional barrier to social integration (García-Segura, 2017), but our results do not confirm this association. In this regard, the absence of significant effects in our model could indicate that other factors, such as the educational context or access to institutional support networks, moderate the influence of these variables on social isolation. Nevertheless, future research should explore these mechanisms in greater depth to better understand the factors that truly contribute to the perception of social isolation in different populations.

Despite theoretical expectations, no significant interaction effects were observed between the moderating variables (gender, cultural origin, and academic performance) and the main predictors. This absence of interaction may be explained, first, by sampling limitations, such as the small size of some subgroups, which reduces statistical power to detect subtle effects (Maxwell et al., 2018). Likewise, the low variability between groups in terms of perceived isolation levels may have reduced the detectable differences (Field, 2018 cited in Kyriazos and Poga, 2023). Another possible explanation is that the interaction effects are small or non-linear, and therefore traditional linear models such as ANCOVA may not have adequately captured them (Hayes, 2018). Finally, it is possible that unaccounted factors, such as institutional support or previous experiences of discrimination, play a more relevant role in moderating the effects of acculturation and language proficiency, as suggested by previous studies in intercultural contexts (Berry et al., 2002; Ward and Geeraert, 2016; Ward and Szabó, 2019).

In light of the findings, several culturally relevant interventions are recommended to reduce social isolation among Indigenous university students. First, instead of solely promoting linguistic assimilation, intercultural bilingual education programs could be developed to recognize and integrate Quechua and Aymara languages in academic and institutional spaces, in accordance with constitutional rights. Second, cultural mentorship initiatives, where advanced Indigenous students support newcomers, may foster psychological acculturation and community cohesion. Third, peer-led support networks can serve as safe spaces for dialogue, identity affirmation, and mutual assistance. These strategies should aim not only to integrate students into the dominant culture but also to transform university environments into truly intercultural spaces that validate linguistic and cultural diversity.

This study has some limitations that should be acknowledged regarding the instruments and methodological design. First, the cross-sectional design limits the ability to establish causal relationships among the variables. Second, the sample was composed exclusively of university students from a specific geographical region and cultural background, which may restrict the generalizability of the findings to other populations. Additionally, the use of snowball sampling, while appropriate for reaching Indigenous students in hard-to-access academic contexts, may introduce selection bias by overrepresenting individuals with greater social connectivity. This non-probabilistic recruitment strategy limits the extent to which the results can be generalized to the broader Indigenous university student population. This study has some limitations that should be acknowledged regarding the instruments and methodological design. First, the cross-sectional design limits the ability to establish causal relationships among the variables. Second, the sample was composed exclusively of university students from a specific geographical region and cultural background, which may restrict the generalizability of the findings to other populations. Additionally, the use of snowball sampling, while appropriate for reaching Indigenous students in hard-to-access academic contexts, may introduce selection bias by overrepresenting individuals with greater social connectivity. This non-probabilistic recruitment strategy limits the extent to which the results can be generalized to the broader Indigenous university student population. Moreover, the study relied partially on self-reported data (PAS-10 and CAS), which may be subject to social desirability bias or individual misinterpretations. This is particularly relevant when assessing constructs such as psychological acculturation and social isolation, where subjective experiences play a central role. Lastly, given the cultural and linguistic diversity of the participants, there may have been variations in the interpretation of certain items, even with prior contextual and linguistic adaptations. These potential cultural nuances should be considered when interpreting the results. Furthermore, although the CAS was originally designed for adolescent populations, its structure proved suitable for evaluating social isolation in university students in this context. Nonetheless, future studies should continue to assess its validity and reliability in diverse academic and cultural settings to confirm its broader applicability. Expanding the use of culturally adapted instruments such as the CAS could contribute to a deeper understanding of the multifaceted experiences of isolation in higher education environments.

## Data Availability

The raw data supporting the conclusions of this article will be made available by the authors, without undue reservation.
