# Supplementary material for: Social isolation among indigenous college students in Peru: the role of language, culture, and acculturation
Source: Front Sociol. 2025 Jun 23;10:1597952. doi: 10.3389/fsoc.2025.1597952 (PMC12230006; doi:10.3389/fsoc.2025.1597952)
Supplement: Supplementary file 1 [file Data_Sheet_1.pdf]

Escala de Aculturación Psicológica (PAS-10)

Le agradecemos por participar en este estudio que busca comprender cómo las personas, en especial de origen quechua o aymara, experimentan el proceso de adaptación psicológica al ingresar a un entorno cultural y educativo distinto.

Este cuestionario tiene como finalidad conocer sus pensamientos y emociones en relación con este proceso de cambio cultural. Las preguntas que se presentan a continuación abordan aspectos cognitivos (cómo ha cambiado su forma de pensar) y emocionales (cómo se ha sentido durante este proceso de adaptación). Por favor, responda con sinceridad cada afirmación, marcando la opción que mejor refleje su experiencia personal: 1 (En desacuerdo), 2 (Ni de acuerdo ni en desacuerdo), 3 (De acuerdo). Por favor, marque la casilla correspondiente junto a cada afirmación.

Sus respuestas son completamente anónimas y se identificarán únicamente con un código asignado.

Procedencia cultural:

☐ Quechua ☐ Aymara

	**1**<br>**En desacuerdo**	**2**<br>**Ni de acuerdo ni en desacuerdo**	**3**<br>**De acuerdo**
1. He notado cambios en mis patrones de pensamiento desde que me adapté a la nueva cultura.	☐	☐	☐
2. Soy capaz de entender y asimilar las normas culturales de mi entorno actual.	☐	☐	☐
3. Mi identidad cultural ha experimentado cambios desde que me integré a esta nueva cultura.	☐	☐	☐
4. Valoro la importancia de comprender y adoptar las costumbres y creencias de la cultura anfitriona.	☐	☐	☐
5. Participo en un aprendizaje constante buscando conocer la	☐	☐	☐

historia, los valores y las tradiciones de la cultura anfitriona.			
6. Siento nostalgia por mi cultura de origen cuando estoy inmerso en la cultura anfitriona.	☐	☐	☐
7. Me siento emocionalmente conectado con otras personas en este nuevo entorno cultural.	☐	☐	☐
8. Estoy emocionalmente satisfecho con mi adaptación a la nueva cultura.	☐	☐	☐
9. Me siento entusiasta ante las diferencias culturales.	☐	☐	☐
10. Me siento motivado a conocer más sobre la cultura anfitriona.	☐	☐	☐

ENGLISH VERSION:

Psychological Acculturation Scale (PAS-10)

Thank you for taking part in this study, which seeks to understand how individuals—especially those of Quechua or Aymara origin—experience the process of psychological adaptation when entering a different cultural and educational environment.

This questionnaire aims to explore your thoughts and emotions related to this cultural transition. The following statements address both cognitive aspects (how your way of thinking has changed) and emotional aspects (how you have felt during this adaptation process).

Please respond sincerely to each statement by marking the option that best reflects your personal experience: 1 (Disagree), 2 (Neither agree nor disagree), 3 (Agree). Check the appropriate box next to each item.

Your responses are completely anonymous and will be identified only by a code.

Cultural background:

☐ Quechua ☐ Aymara

	**1<br>Disagree**	**2<br>Neither agree nor disagree**	**3<br>Agree**
1. I have noticed changes in my thinking patterns since adapting to the new culture.	☐	☐	☐
2. I am able to understand and assimilate the cultural norms of my current environment.	☐	☐	☐
3. My cultural identity has undergone changes since I moved into this new culture.	☐	☐	☐
4. I value the importance of understanding and adopting the customs and beliefs of the host culture.	☐	☐	☐
5. I engage in ongoing learning by seeking knowledge about the history, values, and traditions of the host culture.	☐	☐	☐
6. I experience nostalgia for my culture of origin when immersed in the host culture.	☐	☐	☐

7. I feel emotionally connected with others in this new cultural environment.	☐	☐	☐
8. I am emotionally satisfied with my adaptation to the new culture.	☐	☐	☐
9. I feel enthusiastic about cultural differences.	☐	☐	☐
10. I feel motivated to learn about the host culture.	☐	☐	☐
