# Supplementary material for: Social isolation among indigenous college students in Peru: the role of language, culture, and acculturation
Source: Front Sociol. 2025 Jun 23;10:1597952. doi: 10.3389/fsoc.2025.1597952 (PMC12230006; doi:10.3389/fsoc.2025.1597952)
Supplement: Supplementary file 2 [file Data_Sheet_2.pdf]

Spanish Language Proficiency Rubric (CEFR-Based)

This rubric was used to evaluate the Spanish L2 proficiency of Indigenous university students from Quechua and Aymara backgrounds. The assessment focused on CEFR levels A2 to B2 and was conducted in approximately 20 minutes per participant. The evaluation covered four language skills: listening, reading, speaking, and writing. Below are selected CEFR performance descriptors adapted for this population.

Listening Comprehension

CEFR Level	Descriptor
A2	Can understand phrases and expressions related to areas of most immediate priority (e.g., basic personal and academic information).
B1	Can understand the main points of clear standard speech on familiar matters related to university and student life.
B2	Can understand extended speech and lectures and follow complex lines of argument provided the topic is reasonably familiar.

Reading Comprehension

CEFR Level	Descriptor
A2	Can read very short, simple texts. Can find specific, predictable information in simple everyday material.
B1	Can understand texts that consist mainly of high frequency university-related language.
B2	Can read articles and reports concerned with contemporary problems in which the writers adopt particular stances or viewpoints.

Speaking

CEFR Level	Descriptor
A2	Can communicate in simple and routine tasks requiring a simple and direct exchange of information.
B1	Can deal with most situations likely to arise whilst interacting in an academic environment.

B2	Can interact with a degree of fluency and spontaneity that makes regular interaction with native speakers quite possible.

Writing

CEFR Level	Descriptor
A2	Can write short, simple notes and messages relating to matters in areas of immediate need.
B1	Can produce simple connected text on familiar topics or topics of personal interest.
B2	Can write clear, detailed text on a wide range of subjects related to academic and personal interest.
